# PAD4 is not essential for disease in the K/BxN murine autoantibody-mediated model of arthritis

**DOI:** 10.1186/ar3829

**Published:** 2012-05-02

**Authors:** Amanda S Rohrbach, Saskia Hemmers, Sanja Arandjelovic, Maripat Corr, Kerri A Mowen

**Affiliations:** 1 Department of Chemical Physiology, The Scripps Research Institute, 10550 North Torrey Pines Road, La Jolla, California, 92037, USA; 2 Department of Medicine, University of California, San Diego, 9500 Gilman Drive, La Jolla, California, 92093, USA

## Abstract

**Introduction:**

Both murine and human genome-wide association studies have implicated peptidyl arginine deiminase (PAD4) as a susceptibility gene in rheumatoid arthritis (RA). In addition, patients with RA commonly have autoantibodies which recognize PAD4 or and/or citrullinated peptides. This study aims to evaluate the role of PAD4 in the effector phase of arthritis.

**Methods:**

PAD4 knock out (KO) and wild type (WT) C57BL/6J mice were injected with K/BxN sera to induce disease. Progression of disease was monitored by measuring paw and ankle swelling and clinical indexes of disease, and pathogenesis was assessed by indexing of clinical progression on paws collected from WT and PAD4 KO mice injected with K/BxN serum. PAD4 activity was determined by visualization of neutrophil extracellular traps (NETs) and immunohistological analysis of histone citrullination.

**Results:**

PAD4 activity is readily detectable in the inflamed synovium of WT but not PAD4 deficient animals, as demonstrated by histone citrullination and NET formation. However, PAD4 WT and KO animals develop K/BxN serum transfer disease with comparable severity and kinetics, with no statistically significant differences noted in clinical scores, swelling, joint erosion or joint invasion.

**Conclusions:**

PAD4 WT and KO mice develop disease in the K/BxN serum transfer model of arthritis with similar severity and kinetics, indicating that PAD4 is dispensable in this effector phase model of disease.

## Introduction

Citrulline-containing proteins are generated through posttranslational modification of arginine residues in a reaction catalyzed by the Ca^2+^-dependent peptidyl arginine deiminases (PADs). There are five PAD family members, but only PAD2 and PAD4 expression are closely linked with inflammation in RA synovial tissue [1,2]. While PAD2 is broadly expressed across tissue types, including by immune cells, PAD4 exhibits an expression pattern restricted to immune cell types, in particular macrophages and granulocytes [1,3].

Rheumatoid arthritis (RA) is a chronic autoimmune disease characterized by systemic inflammation, chronic synovitis, joint destruction and bone loss, affecting approximately 2% of the world population [4]. Plasma and synovial biopsy specimens from patients with RA contain high levels of citrullinated proteins [5,6], and anti-citrullinated peptide antibodies (ACPAs) exhibit high specificity and sensitivity as diagnostic markers of the disease [7]. Anti-citrulline peptide antibodies can appear before disease onset and correlate with the most erosive form of RA [8].

PAD4 shows elevated expression in RA [1,9], and RA patients generate high affinity anti-PAD4 autoantibodies which correlate with more severe disease [10-12]. Further, variants of PAD4 are linked to RA in several Japanese and Korean cohorts, although this association has not held up in most North American and European study groups, despite the prevalence of ACPA in all ethnic groups [13]. Thus, the development of autoantibodies to citrullinated epitopes and PAD4 and elevated PAD4 expression in RA, suggests that aberrant PAD activity may contribute to disease pathogenesis.

The offspring of an intercross between the KRN TCR transgenic mouse specific for a bovine RNase (42-56) in the context of I-A^k ^and the I-A^g7^-expressing non-obese diabetic (NOD) background, spontaneously develop a progressive, inflammatory joint disease with features similar to human RA (K/BxN mice) [14]. The autoantigen in this model is glucose-6-phosphate isomerase (GPI), a ubiquitous cytoplasmic enzyme [15]. Treatment with the sera of K/BxN mice or purified anti-GPI autoantibodies is sufficient to transfer disease to healthy animals, even in animals devoid of B and T cells [14,15]. Because autoantibodies are passively transferred, this model focuses on immune recruitment and joint destruction (effector phase), rather than the breaking of immune tolerance (priming phase). Innate immune signals are critical for this model because mice deficient in the alternative complement pathway, the C5a receptor, the CXCR2 chemokine receptor, interleukin-1 receptor (IL-1R), and myeloid differentiation primary response protein (MyD88) are resistant to disease [14,16,17]. Further, arthritis was not sustained in toll-like receptor 4 (TLR4) mutant mice [16]. Passively transferred arthritis also requires the presence of mast cells and neutrophils [18-21].

Neutrophils are among the first immune cell types to accumulate during an inflammatory response [22]. In response to inflammatory stimuli, neutrophils decondense their chromatin and actively expel their DNA-producing neutrophil extracellular traps (NETs) that are decorated with granular and nuclear proteins, including citrullinated histones [23,24]. Incubation of neutrophils with phorbol 12-myristate 13-acetate (PMA), hydrogen peroxide, lipopolysaccharide (LPS), bacteria, and yeast induces NET formation [24-27]. Our lab and others have shown that PAD4 is essential for the production of NETs and NET-associated histone citrullination [24,25,27]. PAD4-mediated histone citrullination is thought to play a mechanical role in NET formation, where the conversion of positively charged arginine residues into the neutral citrulline amino acid by PAD4 promotes chromatin decondensation [24]. PAD4-mediated NET formation is critical for controlling at least a subset of bacterial infections because PAD4-deficient mice are more susceptible to infectious disease in a necrotizing fasciitis model [27].

NET formation, although critical for the full activation of the innate immune response [27], has also been implicated in inflammatory disease pathogenesis, including the autoimmune disorder lupus [28], cystic fibrosis ([29-31], sepsis [32], and thrombosis [33]. Interestingly, it has been suggested that NETs offer a possible mechanism by which PAD4 may be liberated from the cell to generate citrullinated antigens and exacerbate inflammation [9,34]. Recently, Dwievedi *et al. *described hypercitrullination in neutrophils from arthritic patients, as well as the specific reactivity of arthritic serum to activated neutrophils and citrullinated histones [34]. It is unclear whether PAD4-induced NET formation plays a role in the RA inflammatory process.

Recently, using a genome-wide screen of mice, Johnsen *et al. *identified the region containing *Padi4 *as being putatively involved in the development of K/BxN arthritis and demonstrated that increases in the transcripts of both PAD2 and PAD4 correlates with increased severity of disease [35]. Because the K/BxN model is neutrophil dependent, PAD4 activity is required for NET formation and PAD4 overexpression correlates with rheumatoid arthritis in patients, we sought to determine the extent to which PAD4 would contribute to the effector phase of arthritis using the K/BxN-serum transfer model [20,25,27,36].

## Materials and methods

### Mice

PAD4 knockout (KO) mice were generated as previously described [25]. KRN T cell receptor (TCR) transgenic mice were a gift from Drs D Mathis and C Benoist (Harvard Medical School, Boston, MA) and the Institut de Génétique et de Biologie Moléculaire et Cellulaire (Strasbourg, France) [14]. Wildtype C57BL/6J mice were obtained from the Scripps Research Institute rodent breeding colony. Experiments were reviewed and approved by the Scripps Research Institute Institutional Animal Care and Use Committee.

### Induction of serum-transfer arthritis

K/BxN serum was collected from 6- to 10-week-old K/BxN mice and pooled, and 5- to 6-week-old male PAD4 KO or C57BL/6J WT control mice were injected intraperitoneally (i.p.) with 150 μL pooled K/BxN sera on days 0 and 2. Caliper measurements of paw and ankle swelling were taken daily. Clinical indexes of swelling were assigned according to previously described criteria [37]. Animals were sacrificed at day 5 or day 10 and the hind paws were prepared for fluorescence microscopy or histology.

### Fluorescence Microscopy of Histone Citrullination and NETs

Hind paws were removed from euthanized animals at day 5, post K/BxN serum injection. Ankle joints were exposed and flushed with Roswell Park Memorial Institute media (RPMI) with 0.5% BSA. Peripheral blood was collected by eye bleed. Erythrocytes were lysed in 10 mM KHCO_3_, 150 mM NH_4_Cl NS 1 mM EDTA and remaining cells were washed with PBS. Ankle or peripheral blood cells were attached to poly-lysine-coated glass coverslips and fixed in 4% paraformaldehyde (PFA) with 1% Nonidet P-40 (NP-40) and 0.5% Triton X-100 in PBS. Slides were blocked with 2.5% BSA and 5% goat serum in PBS with 0.5% Tween-20. Citrullinated histones were detected with anti-H4Cit3 (Millipore, Billerica, MA, USA) and goat anti-rabbit Alexa488 (Life Technologies, Grand Island, NY, USA). DNA was labeled with DAPI (Sigma, St. Louis MO, USA). Coverslips were mounted with Prolong Gold Antifade reagent (Life Technologies, Grand Island, NY, USA).

### Histology

Hind paws were removed from euthanized animals, fixed in 4% paraformaldehyde in PBS overnight and decalcified in 0.375 M EDTA pH 7.5 for 3 weeks. Paws were embedded in paraffin and affixed to slides for immunohistochemistry or pathological analysis by The Scripps Research Institute Histology Core. For immunohistochemistry, paraffin-embedded slices on slides were deparaffinized with xylene and rehydrated. Antigens were unmasked by boiling in PBS pH 7.4. Slides were blocked with 10% normal goat serum and endogenous peroxidases were quenched with 3% peroxide in methanol. Sections were then incubated with anti-H4cit3 or control rabbit immunoglobulin (IgG) antibodies (Millipore, Billerica, MA, USA). Labeling was detected with a biotinylated anti-rabbit secondary antibody (Life Technologies, Grand Island, NY, USA), Vectastain Elite ABC reagent (Vector Labs, Burlingame, CA, USA) and DAB (Life Technologies, Grand Island, NY, USA). Slides were counterstained with Shandon Harris hematoxylin (Thermo Scientific, Waltham, MA, USA). Histopathological scoring was performed blinded as described previously [16]. Briefly, ankles of arthritic mice were given scores of 0 to 5 for inflammation, according to the following criteria: 0 = normal; 1 = minimal infiltration of inflammatory cells in the periarticular area; 2 = mild infiltration; 3 = moderate infiltration; 4 = marked infiltration; and 5 = severe infiltration. Knees of arthritic mice were given scores of 0 to 5 for bone resorption, according to the following criteria: 0 = normal; 1 = minimal (small areas of resorption, not readily apparent on low magnification); 2 = mild (more numerous areas of resorption, not readily apparent on low magnification, in trabecular or cortical bone); 3 = moderate (obvious resorption of trabecular and cortical bone, without full-thickness defects in the cortex; loss of some trabeculae; lesions apparent on low magnification); 4 = marked (full-thickness defects in cortical bone and marked trabecular bone loss; without distortion of the profile of the remaining cortical surface) and 5 = severe (full-thickness defects in the cortical bone and marked trabecular bone loss with distortion of the profile of the remaining cortical surface).

## Results

### NETs are found in the joints of K/BxN serum-transfer animals

Neutrophils are the predominant infiltrating cell type in the K/BxN serum transfer arthritis model and are found within synovium throughout the serum-transfer during the course of arthritis disease [17,20]. Importantly, PAD4 is highly expressed in the granulocyte lineage, including neutrophils [3] and is known to be required for the formation of Neutrophil Extracellular Traps (NETs) [25,27]. NETs have been shown to occur in several inflammatory disease states, including lupus [28], sepsis [32,33] and cystic fibrosis [29-31] and are thought to serve as a means for PAD4 to enter into the extracellular space, where it may citrullinate substrates and exacerbate inflammation [9]. NETs are common to several other inflammatory disease states, but to our knowledge, NET structures, *per se*, have not been reported in patients with RA or in murine models of disease. However, recently, Dwivedi *et al. *reported that serum IgGs from patients with Felty's syndrome react to NETs and preferentially to citrullinated histones over unmodified histones, indicating that NET markers, and thus NETs, may be present in Felty's syndrome [34]. Since ACPA and anti-PAD4 antibodies are frequently found in patients with RA [9], it is presumed that aberrant PAD activity influences the priming phase of arthritis. However, it is unknown where PAD4 may also regulate the effector phase of arthritis. We therefore considered whether PAD4-mediated NET formation could contribute to K/BxN-induced inflammation.

**Figure 1:**
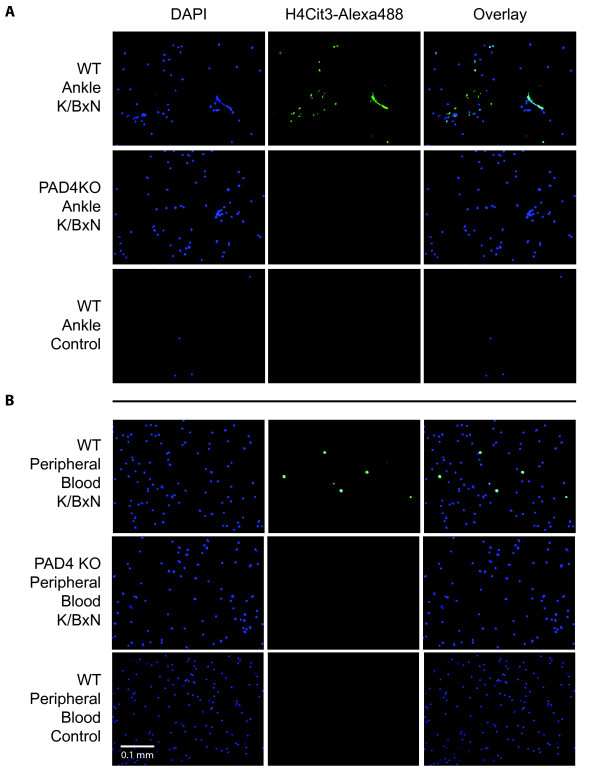
**Wild type (WT) K/BxN serum transfer mice generate synovial neutrophil extracellular traps (NETs)**. Mice were injected with K/BxN serum on days 0 and 2 and sacrificed on day 5. Synovial cells were flushed from the ankle joint (**A**) and peripheral blood was collected from the venous plexus of arthritic or healthy mice (**B**) and stained with antibodies to citrullinated histone H4 and 4',6-diamidino-2-phenylindole (DAPI). Histone citrullination and NETs were readily detectable in the arthritic joints of WT mice, but were not observed in peptidyl arginine deiminase 4 knockout (PAD4 KO) K/BxN mice or WT healthy controls (**A**). Histone citrullination was present in the peripheral blood of WT K/BxN mice, however, no NET formation was observed. Images were taken at 80× magnification and are representative of at least three independent experiments with two mice per experimental group.

To determine whether NET formation occurs in the K/BxN serum transfer model, fluorescence microscopy of flushed synovial cells was performed. PAD4 WT and KO mice were injected with K/BxN serum on days 0 and 2. Mice were sacrificed at day 5 to collect synovial and peripheral blood cells. Cells flushed from the ankles of arthritic WT mice displayed citrullinated histones, a hallmark of PAD4 activity, and NET formation, while those from PAD4 KO mice exhibited no detectable histone citrullination or NET formation, as detected by anti-H4Cit3 and 4',6-diamidino-2-phenylindole (DAPI) (Figure 1A). As expected, few cells could be flushed from the ankles of WT control mice, and none were observed to have citrullinated histones or to form NETs (Figure 1A). Histone citrullination, but not NET formation, was detected in the peripheral blood of WT K/BxN mice, but not WT control mice (Figure 1B). Neither histone citrullination nor NET formation was found in control mice of either genotype (Figure 1B). Thus, NET formation does occur in the K/BxN model of the effector phase of RA and is specific for the arthritic ankle.

### There is abundant PAD4 activity during K/BxN serum-transferred arthritis

**Figure 2:**
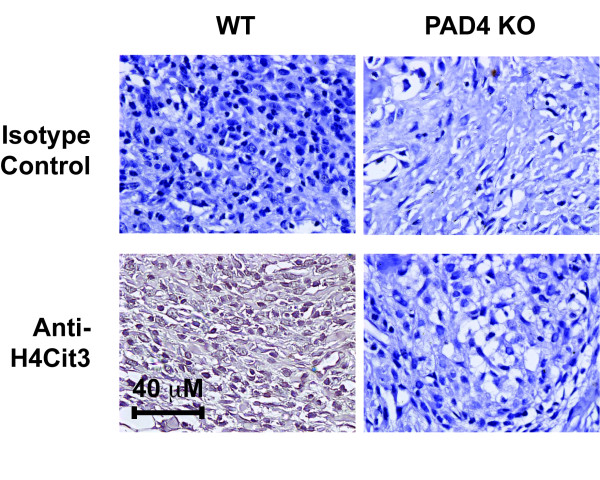
**Peptidyl arginine deiminase 4 **(**PAD4) activity is detected in K/BxN arthritic legs in wild type (WT) but not PAD4 knockout (KO) mice**. PAD4 activity was measured by immunohistochemical detection of citrullinated histone H4 (H4Cit3). WT and PAD4 KO mice were immunized with pooled K/BxN serum as described. Mice were sacrificed 10 days post injection (p.i.) and their legs prepared for histology. Sections were blocked in goat serum and probed with either anti-H4Cit3 antibody or isotype control. Images were taken at 80× magnification. Specific staining of citrullinated histone H4 was apparent on WT but not PAD4 KO slides and is representative of at least 4 independent experiments.

PAD4 is found in the synovial fluid and infiltrating synovial cells of patients with RA [9,38,39]. In the K/BxN serum transfer model, elevated expression of PAD4 in the spleen correlates with the severity of arthritis [35]. To determine whether PAD4 enzymatic activity was a hallmark of the effector phase of arthritis, immunohistochemistry of the PAD4-dependent citrullinated histone H4 was performed [25,27]. Mice were injected with K/BxN serum on days 0 and 2. On day 10, mice were sacrificed, hind limbs were harvested and prepared for histology, and tissue sections were immunostained for citrullinated histone H4. WT mice displayed robust histone H4 citrullination, with the most prominent staining in infiltrating cells within the synovial sublining, which were most likely neutrophils. This modification was dependent upon PAD4 because PAD4 KO mice exhibited no staining for citrullinated histone H4 (Figure 2). These data demonstrate that robust PAD4 activity is present within the ankle joint during the effector phase of arthritis.

### PAD4 deficiency does not protect against disease in serum-transferred arthritis

**Figure 3:**
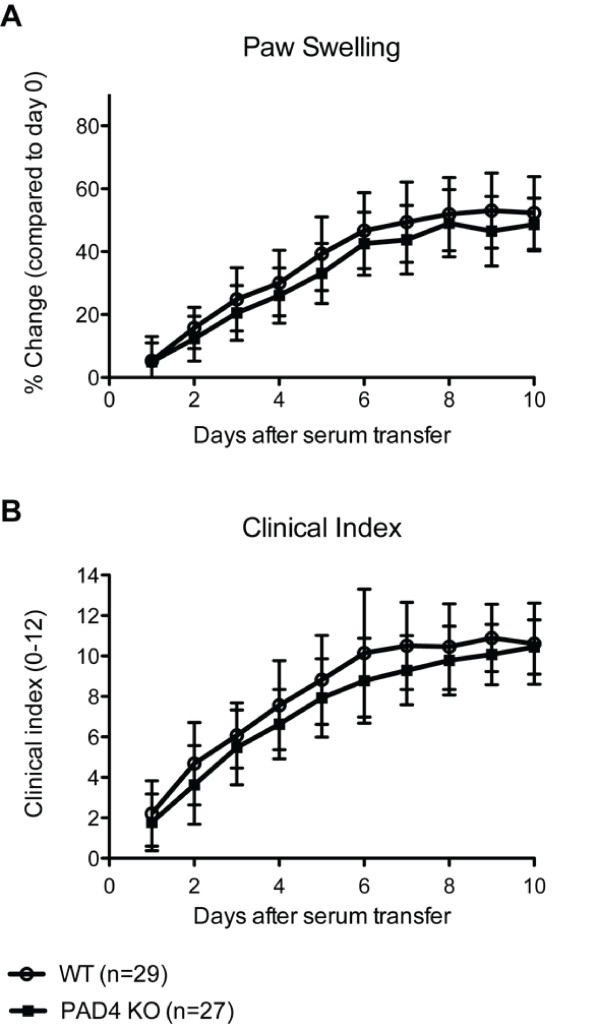
**Progression of K/BxN serum transfer arthritis in peptidyl arginine deiminase 4 **(**PAD4) wild type (WT) (*n *= 29) and knockout (KO) (*n *= 27) mice**. WT and PAD4 KO mice were immunized with pooled K/BxN serum as described and monitored for signs of disease severity for 10 days. Paw swelling (**A**) is expressed as the average percent size increase of four paws and two ankles compared to day 0 measured by caliper. Clinical indexes (**B**) of 0 to 3 were assigned for each paw as previously described [37]. Data are combined from three independent experiments and represented as the average ± standard error of the mean (SEM). No statistically significant difference in the progression of K/BxN disease was apparent between WT and PAD4 KO mice.

To test the importance of PAD4 in the effector phase of arthritis, we injected K/BxN serum into age-matched WT and PAD4 KO C57BL6/J mice on days 0 and 2 and monitored disease progression. Measurements of paw swelling and clinical disease index were taken daily for ten days. Paw swelling was calculated as the percentage increase in paw size compared to day 0. The onset of disease and the overall kinetics of disease were similar between WT and PAD4 KO mice (Figure 3). Although there was a trend toward reduced swelling and lower disease scores in PAD4 KO mice, the differences were small and did not reach statistical significance (Figure 3). Disease is transient in the serum transfer model. We found that PAD4 KO mice recovered with similar kinetics to WT mice (data not shown). Thus, PAD4 is not required for the development or maintenance of K/BxN-serum transferred arthritis.

### Comparable histological features between arthritic WT and PAD4-deficient mice

**Figure 4:**
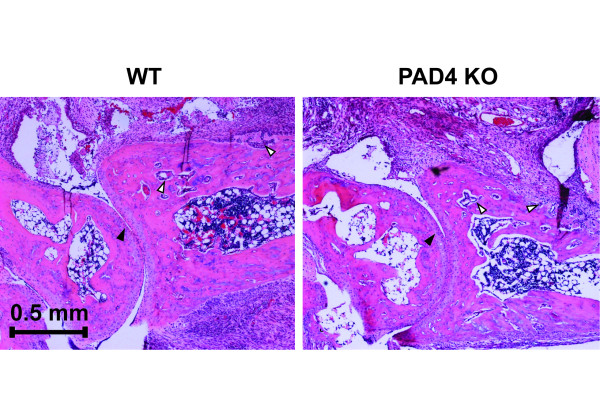
**Pathological evaluation of immune invasion and joint destruction in wild type (WT) and peptidyl arginine deiminase 4 knockout (PAD4 KO) K/BxN mice**. WT (*n *= 27) and PAD4 KO (*n *= 29) animals were immunized with pooled K/BxN serum as described, sacrificed at day 10 post injection (p.i.) and their legs prepared for histology. Hematoxylin- and eosin-stained stained slides were scored blinded by a trained observer for immune cell invasion (0 to 5) and joint erosion (0 to 5). Pictures were taken at 40× magnification and are representative of the average scores for invasion (4) and erosion (2), shown in Table 1. Areas of immune cell invasion into cartilage of joint (closed arrows) and bone reabsorption (open arrows) are highlighted.

Although WT and PAD4 KO mice exhibited a similar level of inflammation, the degree of joint destruction and inflammatory infiltrates could be affected by PAD4 deficiency. Thus, hind limbs were harvested from WT and PAD4 KO mice on day 10 following serum transfer. Histological evaluation by an investigator blinded to the animal genotype for degree of inflammation and erosion of bone was performed. Inflammation and bone erosions were quantified by scoring joint sections on a 0 to 5 scale. Hematoxylin and eosin stain (H&E) sections of the ankle joints revealed a comparable, moderate degree of inflammatory cell infiltration in WT and PAD4 KO mice (Figure 4 and Table 1). In healthy mice, the synovium consists of a single layer. Both WT and PAD4 KO mice developed a proliferative synovial response following K/BxN serum treatment. Furthermore, WT and PAD4 KO mice exhibited a similar level of bone erosion. Therefore, the histological features of the K/BxN serum-mediated model of arthritis are not altered in the absence of PAD4.

**Table 1:** Clinical scores of inflammation and joint erosion in wild-type and peptidyl arginine deiminase 4 knockout K/BxN mice

Group	Inflammation (0 to 5) (mean ± STDEV)	Joint Erosion (0 to 5) (mean ± STDEV)
WT	3.4 ± 1.3	2.5 ± 1.4
PAD4 KO	3.6 ± 1.0	2.2 ± 1.7

Pathological scoring of immune invasion and joint destruction in wild type (WT) and peptidyl arginine deiminase 4 knockout **(**PAD4 KO) K/BxN mice. WT (*n *= 27) and PAD4 KO (*n *= 29) animals were immunized with pooled K/BxN serum as described, sacrificed at day 10 post injection (p.i.), and their legs prepared for histology. Hematoxylin- and eosin-stained slides were scored blinded by a trained observer for immune cell invasion (0 to 5) and joint erosion (0 to 5). Results are represented as the average of 27 and 29 mice per genotype ± standard deviation. Average scores for joint invasion and erosion were similar in WT and PAD4 KO mice.

## Discussion

In this study, we find that the PAD4 enzyme is active within the joint tissue of arthritic mice and leads to NET formation. However, the disease course and histological features of the arthritic joints following K/BxN serum injection were comparable between WT and PAD4-deficient mice, demonstrating that PAD4 is not required for the effector phase of arthritis. Our data are consistent with the findings by Willis *et al*., showing that the PAD inhibitor Cl-amidine provides therapeutic benefit in the collagen-induced arthritis model but has no benefit when arthritic disease is induced by the administration of anti-collagen antibodies. Collagen induced arthritis and injection of anti-collagen antibodies represent the priming and effector phases of disease, respectively. We have now shown that PAD4 is active but not required for disease, in another model of the effector phase, the K/BxN serum transfer model, which is consistent with the finding that PAD inhibitors have no effect on disease when induced by administration by anti-collagen antibodies [40]. Interestingly, Cl-amidine preferentially inhibits PADs 1 and 4 over PAD3, and its effects have not been reported for PADs 2 or 6 [41].

Indeed, Johnsen *et al. *identified the *Padi *locus, which contains the PAD4 gene along with the genes for PADs 1, 2, 3, and 6, as being linked with disease in the K/BxN serum transfer model [35]. PAD2 and PAD4 are the most likely candidates to regulate the effector stage of arthritis as they are both expressed in immune cell types, whereas the expression of PADs 1, 3, and 6 are restricted to the epidermis, hair follicle, and oocyte, respectively. Indeed, increased splenic expression of both PAD2 and PAD4 correlated with disease severity in the K/BxN model [35]. Further, within the *PAD *region, a SNP found within the *Padi2 *locus showed the most significant association with disease [35]. We speculate that the loss of PAD2 and PAD4 together may produce a more apparent phenotype in the K/BxN model [40]. PAD2 KO mice have been reported [42], however the PAD2 and PAD4 loci are approximately 1 centimorgan apart, and therefore, the recombination frequency between the targeted PAD2 and PAD4 alleles would be quite small, making the generation of *Padi2/Padi4 *DKO mice unlikely. While our data demonstrate that PAD4 is not required for the development of the K/BxN serum-transfer model, it is possible that there might be redundant activity of other PADs or they may independently contribute to the pathogenesis of antibody-mediated arthritis.

The blood of RA patients contains autoantibodies directed against a number of self-antigens. Many autoantibodies in RA are directed against citrullinated proteins. In fact, the presence of anti-citrulline antibodies is a better predictor of RA than rheumatoid factor [43]. Variants of PAD4 are linked to RA in several Japanese and Korean cohorts, and the mRNA of a disease-associated allele is more stable than a non-disease associated allele [36,44]. It has been proposed that PAD4 is linked to RA because PAD4 citrullination of peptides leads to a breakdown in tolerance to self-antigens [43,45]. In support of this, treatment of mice with the PAD inhibitor Cl-amidine in the collagen-induced arthritis (CIA) model reduces the levels of citrulline found in the serum and synovial tissue, diminishes the formation of autoantibodies, and ameliorates disease [40]. Thus, it will be interesting to determine whether the effects of Cl-amidine are attributable to PAD4 activity by examining the susceptibility of PAD4 KO mice to arthritic disease using the CIA model.

Incubation of human neutrophils with lipopolysaccharides (LPS), TNFα, N-formyl-methionine- leucine-phenylalanine (fMLP), or lipoteichoic acid and murine neutrophils with LPS or bacteria, has been shown to induce histone deimination and NET formation, marking PAD4 activity [24-27]. Further, we detected deiminated histone H4 in lung leukocytes isolated from influenza-infected mice [25]. In this report, we find that PAD4 activity is readily detected within the affected arthritic joint. In WT mice receiving K/BxN serum, the presence of deiminated histones corresponded primarily to the infiltrating cells of the joint sublining, which is consistent with the expression pattern of PAD4 found in patients with RA [46,47]. The stimulus that induces PAD activity during autoimmune-mediated inflammation is undefined. However, the LPS receptor TLR4, has been linked to animal models of arthritis, perhaps because of the activation of TLR4 by endogenous ligands, such as Tenascin-C [16,48,49].

NETs possess potent microbicidal capabilities but have also been implicated in chronic inflammatory diseases [23,50]. NET formation is linked to cystic fibrosis [29-31]. Similarly, the formation of NETs contributes to endothelial and tissue injury during sepsis [32]. In autoimmune small-vessel vasculitis, anti-neutrophil cytoplasm antibodies (ANCA) trigger the formation of NETs, promoting necrotic inflammation of the blood vessels [51]. Systemic lupus erythematous (SLE) is a systemic autoimmune disease characterized by the formation of pathogenic immune complexes. When activated by autoantibodies, neutrophils isolated from patients with SLE produce NETs, exposing immunostimulatory proteins and potential autoantigens and leading to the induction of Type I interferons by plasmacytoid dendritic cells [28,52,53]. Collectively, these results support the notion that NET production can contribute to disease pathogenesis in inflammatory conditions. While hypercitrullination of neutrophil histones has been reported in patients with RA [34], it is unclear whether NETs have a role in RA inflammation. Our results suggest that PAD4 activity and subsequent NET formation is present in the K/BxN serum transfer model, but is not required for this model of effector phase of disease.

Our data demonstrate that PAD4 is not necessary for the antibody-dependent, effector stage of arthritis. It is also possible that compensation by other PAD family members, PAD2 in particular, may mask the function of PAD4 in arthritis, although we note that PAD2 expression is not upregulated in PAD4-deficient neutrophils [25]. Finally, since the *Padi *locus is linked to disease severity in the K/BxN serum transfer model, it may be necessary to eliminate several PAD family members, either by targeting multiple locations within the PAD locus or by combining treatment with specific PAD inhibitors with targeted PAD alleles. Further studies will be necessary to dissect the role of PAD4 in the priming phase of arthritis.

## Conclusions

NET formation is known to correlate with inflammatory disease [28,32,33,52,53], however NET formation has not been reported in RA. NET formation is dependent on PAD4 and the association between PAD4 and RA is well-established [9]. In this report, we show that in the K/BxN murine model of arthritis, which emulates the effector phase of disease, PAD4 activity and NET formation are detected. PAD4 activity is not, however, required for disease, as PAD4 WT and KO mice develop K/BxN-induced arthritis with similar severity and kinetics. Overall our data indicate that PAD4 is dispensable in this model. However this does not eliminate a role for PAD4 in the priming phase of disease. Future studies will consider the role of PAD4 in priming phase models of RA, including the CIA model.

## Abbreviations

ANCA: anti-neutrophil cytoplasmic antibodies; CIA: Collagen-induced arthritis; DAPI: 4',6-diamidino-2-phenylindole; fMLP: formyl-methionine-leucine-phenylalanine; H&E: hematoxylin and eosin stain; IgG: immunoglobulin; IL-1R: interleukin-1 receptor; KO: knockout; LPS: lipopolysaccharide; MyD88: myeloid differentiation primary response protein; PAD: Peptidyl arginine deiminase; PMA: phorbol 12-myristate 13-acetate; NET: neutrophil extracellular trap; NOD: non-obese diabetic;RA: rheumatoid arthritis; SLE: systemic lupus erythematous; SNP: single nucleotide polymorphism; TCR: T cell receptor; TLR: toll-like receptor; WT: wild type.

## Competing interests

No financial support or benefits were received from commercial sources for this study. The authors declare no financial interests or conflicts of interest related to this work.

## Authors' contributions

KM conceived the experiments. AR and KM designed the experiments, analyzed data and prepared the manuscript, with input from SA, SH and MC. AR, SA and SH prepared reagents and executed experiments. MC scored the histological samples. The manuscript was read and approved by all authors.
